# The Week's Work

**Published:** 1910-05-14

**Authors:** 


### THE WEEK'S WORK.

### THE KING'S DEATH.

At this moment, when the unhappy event of last week that has plunged the Empire into mourning is still so near to us that the mind cannot grasp all that it means, it is not to be wondered at that in some quarters the full sense of the great loss we have sustained through the death of King Edward VII. has not yet been realised. To hospital workers, however, this calamity comes as a shock, the importance of which it only needs a moment's reflection to feel. The day of the King's death to us, who know his manifold interests in hospital life, in charitable work, and in everything that pertains to the relief of distress and suffering in the realm he ruled so well, is indeed a day of vast solitude, not merely in the sense of polite convention as the Roman historian used the term, but in terribly sad reality. Hospital workers knew on Saturday morning, when they hoisted the flags to half-mast over their institutions, that they had lost not only a great patron but a personal friend, whose interest was always keen and whose sympathy always practical. Elsewhere in this issue we deal more fully with the story of King Edward VII. and the hospitals. Here it is allowable to add that many a hospital chairman, many a hospital secretary knows from personal experience how keen was his late Majesty's interest in the welfare of his sick subjects and in the institutions that were designed for their relief. We reveal no State secrets when we state that during recent years our late King was closely observant of the trend of public opinion with regard to hospitals, and that he followed the developments in institutional progress and the corresponding advances in medical science with careful attention. More and more these developments are trenching upon matters political, and it is unfortunate, in many respects, that where such overlapping occurs the Sovereign's opinions and wishes cannot be expressed with that freedom that would make them valuable guides for the institutional world and the public at large. In all matters of pure charity his late Majesty's work is sufficiently well known to win the cordial appreciation of everyone interested in hospital matters, but it is possible, judging from the comparatively scanty allusions which have been made to it in the lay Press, that the public as a whole does not yet fully grasp the fact that King Edward VII. was one of the greatest hospital workers of his day.

### KING EDWARD AS A HOSPITAL PATRON.

A mere glance at the list of institutions of which he was a patron will show how wide and catholic his interests were. He was the patron of numerous metropolitan and provincial hospitals before he came to the throne, and since 1901 he largely increased his patronage and, aided by his kindly-hearted consort, Queen Alexandra, became the close friend and helper of many other institutions. The fact that he was a patron of a hospital meant much more than that he merely allowed the institution the liberty of using his name to head the list of its well-wishers or of attaching the prefix "Royal" to the title of the charity. It meant that he took a personal interest in the work of that hospital. He gave his patronage to none unworthy of that high distinction, and satisfied himself, in many cases by personal inspection, that the institutions so honoured were carried on in a blameless and thoroughly efficient manner. Nor did he scruple, when necessary, to make it known that without efficiency and proper management no institution could hope to continue under his patronage. Hospital workers will readily call to mind his Majesty's refusal to allow his name to be associated with hospitals whose financial positions were unsound, his withdrawal of the Royal patronage from the old Royal Military Benevolent Fund after a full preliminary investigation into the affairs of that charity, and the more recent instance in which he severed his connection with an educational institution. This active personal interest on the part of Royalty has served as a stimulus and incentive to hospital secretaries and committee men. They knew that the King's patronage was not a careless gift, but a great responsibility, to be retained only so long as the authorities of the charities to which it was granted showed by their action and management that they were worthy to keep it. Undoubtedly that knowledge has helped to keep many a hospital alert, vigorous, and up to date, and in that way perhaps, more than in any other, King Edward VII. accomplished excellent work on behalf of all charitable institutions in his realm, though in other directions his influence upon the institutional world was felt beneficially and characteristically. The establishment of the Prince of Wales' Fund, now the King Edward VII. Hospital Fund, was a monumental work on behalf of charity that has never been equalled, and is unlikely ever to be surpassed. Like his beloved mother, Queen Victoria, he knew the needs of his subjects, and he gave largely and liberally to charity. His contributions, like his patronage, meant far more than the mere amount of money they intrinsically represented, generous though that amount was. They meant that his Majesty the King endorsed and approved the work that the charities were doing, and as such they were brilliant advertisements of the efficiency of the institutions that were privileged to receive them. That our voluntary hospitals to-day are in the flourishing condition in which they undoubtedly are is due largely to King Edward's interest in their welfare, his unfailing sympathy with their work, and the active, generous, and personal support that he gave to them.

### A PERSONAL LOSS.

In the circumstances the formal letter of condolence which every hospital secretary, whose chief authority as patron was our late King, has drafted and sent to Buckingham Palace this week is no mere empty form of phrasing a conventional loyal expression of sympathy. It is a real expression of grief at a loss which is personal in so far that it utters the dominant feeling of poignant regret and sorrow which every hospital worker feels at the death of a friend who did so much for our work, and who sympathised so cordially with our aims and aspirations. Some of us who were privileged to know him personally, who have guided him through the hospital wards and witnessed his eager interest in the daily work of a large institution for the relief of the suffering and sick, will feel that loss all the more deeply; but all who are aware of what he has achieved for hospitals and charity generally will realise that the nation's loss is a great one. The eulogies that have been passed upon the dead King are many and well deserved, but none are more competent to laud King Edward's greatness than those who have had the honour of being associated with him in charitable and institutional work. They above all recognise that Aristotle's definition of a king fitted him eminently, and that St. Basil would have deemed him, as we all deem him, a true king and great and good. Among the mourners that will follow his bier they will have their place, and in sincerity of sorrow and earnestness of regret at his loss will stand second to none.

### THE KING'S DEATH AND THE HOSPITALS.

This sad event puts an end, for the time being, to all hospital functions. Festival dinners, annual meetings, staff reunions, and similar celebrations are postponed indefinitely. There can be no excuse for the authorities of an institution of which his late Majesty was the patron, or of which any of the Royal Family are patrons, to go on with its usual functions so long as the period of prescribed mourning has still to be passed. On all hospitals the flags are draped and at half-mast, and no convivial or general celebration can take place while these signs of mourning are still to be seen. In some cases where an important function has been arranged to take place this month it may be deemed justifiable to go on with it. We have heard it argued, for instance, that his late Majesty would surely not have wished a charity in which he took so much interest to suffer through postponing or cancelling engagements calculated to benefit it. That argument needs no refutation to those who feel the King's death as acutely as hospital men do, and we believe that no institution is likely to suffer financially or otherwise by falling into line with those the authorities of which have already, without loss of time and with a thoughtfulness and sincerity that show how deeply they feel this calamity, cancelled important functions which had been fixed for the present month. We feel confident that hospital committees will see the matter in its proper light, and act accordingly.

### THE FUTURE.

A word is permissible in conclusion as to the future. We have lost a good friend and a great King, and in the nature of things we have to pause in our grief to hail his successor. King George V. has already shown that he is an active sympathiser with the hospitals, and there is every reason to believe that he will follow in the steps of his grandmother and father, and work as actively and as sympathetically in the cause of charity as he worked when Duke of York and Prince of Wales. His intimate connection as president with many of our institutions will perhaps no longer be possible, but as patron of these charities his support will still be available, and will be as helpful as was his father's. He is known as a hospital worker, whose knowledge of institutional affairs is intimate and, indeed, profound. As a hospital visitor he has proved himself to possess a thorough acquaintance with the needs of a modern institution, and as President of the King's Fund his work on behalf of hospitals has won him the admiration and respect of everyone who takes an interest in the welfare of our charitable institutions. It is, therefore, with a sincere appreciation of the success that has attended his endeavours in the past that we join in the utterance of respectful and loyal congratulation on his accession to the Throne, and in the fervent prayer that all his subjects breathe,

God Save the King!

### THE WEEK'S WORK.

There is, apart from the great event of the week, nothing that calls for special comment in the institutional world. For the moment controversial matters are laid aside, and the items of hospital news that appear in our columns deal largely with the past fortnight and with subjects of immediate importance. We would, however, draw attention to the development in the Bolton Infirmary question. In accordance with the request of the Infirmary Board, referred to in The Hospital of March 12, p. 700, the British Medical Association has nominated Dr. Donald J. Mackintosh, M.V.O., the well-known superintendent of the Glasgow Western Infirmary, and an expert on hospital construction, to visit and report upon the condition and administration of the institution. With this matter we hope to deal more fully in a future issue.

### THE DRUG MARKET

The quietness of the Drug market has this week been accentuated by the King's death, and although business has not actually been suspended, the market has been more or less depressed. There have, however, been several important alterations in prices since the last report appeared. The most important of these is the advance in the price of asafetida; this drug has been sold at double the price recently asked, and at least three times the normal value. So far as can be seen at present the value is not likely to recede, as there is a scarcity of good quality but a steady demand. Ipecacuanha is about 15 per cent. cheaper. Buchu leaves are again dearer and the available supplies are very limited. Chloral hydrate has been reduced in price 50 per cent., but it seems likely that quotations will be advanced again. Carbolic acid is tending slightly dearer, as also is cream of tartar. Cod liver oil is rather lower. Coca leaves have advanced in price somewhat and are in good demand. The demand for quinine has slackened, and castor oil has suffered a slight set-back after the recent advances.

